# Supplementary material for: Ultraviolet-C light at 222 nm has a high disinfecting spectrum in environments contaminated by infectious pathogens, including SARS-CoV-2
Source: PLoS One. 2023 Nov 28;18(11):e0294427. doi: 10.1371/journal.pone.0294427 (PMC10684113; doi:10.1371/journal.pone.0294427)
Supplement: S1 Table — (PPTX) [file pone.0294427.s001.pptx]

## Slide 1
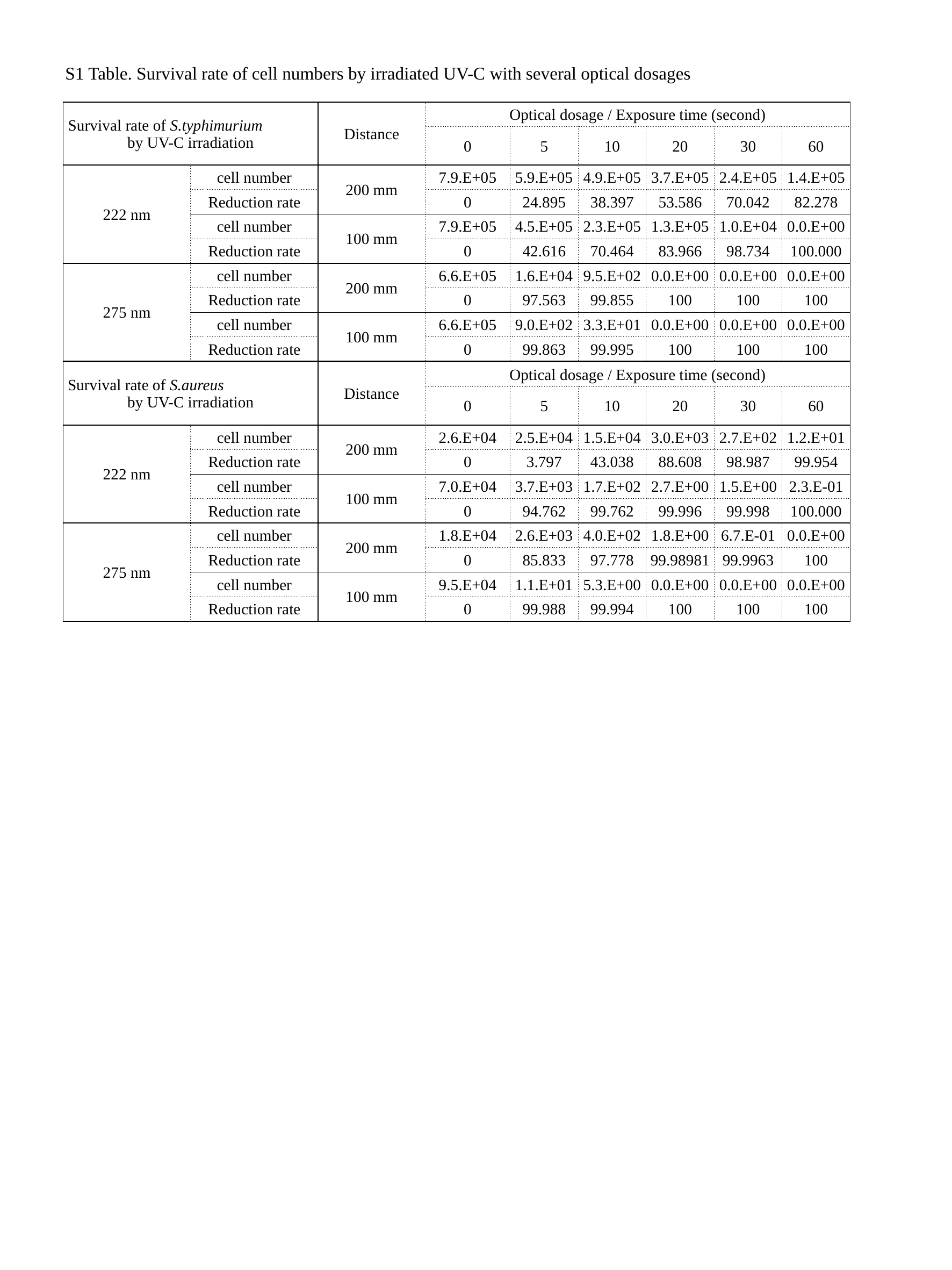

S1 Table. Survival rate of cell numbers by irradiated UV-C with several optical dosages
| Survival rate of S.typhimurium by UV-C irradiation | | Distance | Optical dosage / Exposure time (second) | | | | | |
| --- | --- | --- | --- | --- | --- | --- | --- | --- |
| | | | 0 | 5 | 10 | 20 | 30 | 60 |
| 222 nm | cell number | 200 mm | 7.9.E+05 | 5.9.E+05 | 4.9.E+05 | 3.7.E+05 | 2.4.E+05 | 1.4.E+05 |
| | Reduction rate | | 0 | 24.895 | 38.397 | 53.586 | 70.042 | 82.278 |
| | cell number | 100 mm | 7.9.E+05 | 4.5.E+05 | 2.3.E+05 | 1.3.E+05 | 1.0.E+04 | 0.0.E+00 |
| | Reduction rate | | 0 | 42.616 | 70.464 | 83.966 | 98.734 | 100.000 |
| 275 nm | cell number | 200 mm | 6.6.E+05 | 1.6.E+04 | 9.5.E+02 | 0.0.E+00 | 0.0.E+00 | 0.0.E+00 |
| | Reduction rate | | 0 | 97.563 | 99.855 | 100 | 100 | 100 |
| | cell number | 100 mm | 6.6.E+05 | 9.0.E+02 | 3.3.E+01 | 0.0.E+00 | 0.0.E+00 | 0.0.E+00 |
| | Reduction rate | | 0 | 99.863 | 99.995 | 100 | 100 | 100 |
| Survival rate of S.aureus by UV-C irradiation | | Distance | Optical dosage / Exposure time (second) | | | | | |
| --- | --- | --- | --- | --- | --- | --- | --- | --- |
| | | | 0 | 5 | 10 | 20 | 30 | 60 |
| 222 nm | cell number | 200 mm | 2.6.E+04 | 2.5.E+04 | 1.5.E+04 | 3.0.E+03 | 2.7.E+02 | 1.2.E+01 |
| | Reduction rate | | 0 | 3.797 | 43.038 | 88.608 | 98.987 | 99.954 |
| | cell number | 100 mm | 7.0.E+04 | 3.7.E+03 | 1.7.E+02 | 2.7.E+00 | 1.5.E+00 | 2.3.E-01 |
| | Reduction rate | | 0 | 94.762 | 99.762 | 99.996 | 99.998 | 100.000 |
| 275 nm | cell number | 200 mm | 1.8.E+04 | 2.6.E+03 | 4.0.E+02 | 1.8.E+00 | 6.7.E-01 | 0.0.E+00 |
| | Reduction rate | | 0 | 85.833 | 97.778 | 99.98981 | 99.9963 | 100 |
| | cell number | 100 mm | 9.5.E+04 | 1.1.E+01 | 5.3.E+00 | 0.0.E+00 | 0.0.E+00 | 0.0.E+00 |
| | Reduction rate | | 0 | 99.988 | 99.994 | 100 | 100 | 100 |
