# Supplementary material for: Ultraviolet-C light at 222 nm has a high disinfecting spectrum in environments contaminated by infectious pathogens, including SARS-CoV-2
Source: PLoS One. 2023 Nov 28;18(11):e0294427. doi: 10.1371/journal.pone.0294427 (PMC10684113; doi:10.1371/journal.pone.0294427)
Supplement: S1 Raw image — (PDF) [file pone.0294427.s002.pdf]

Figure 4B. Non-infected

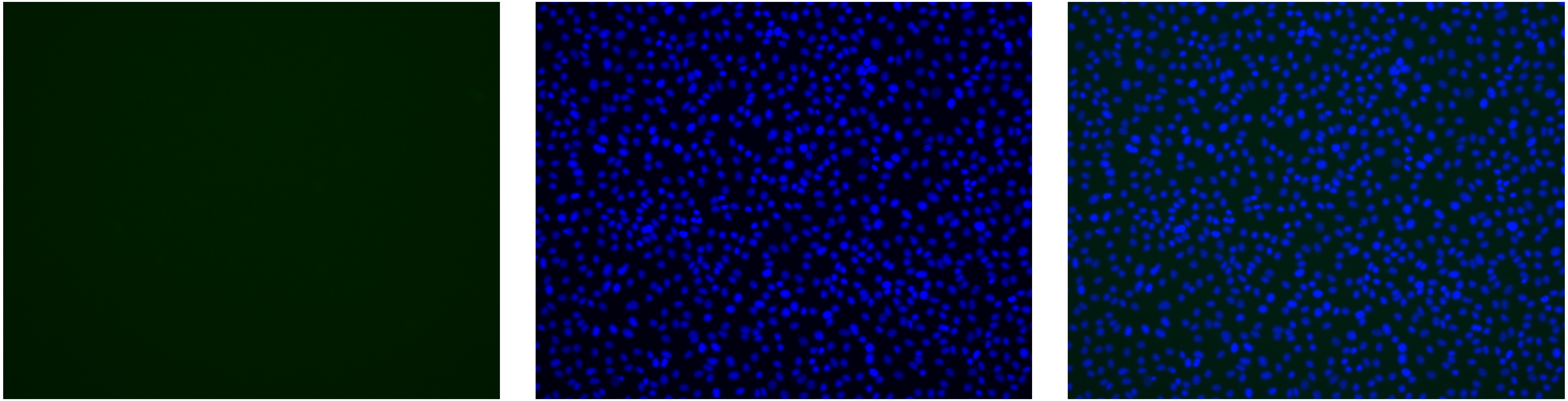

GFP DAPI Overlay

Figure 4B. 0 second

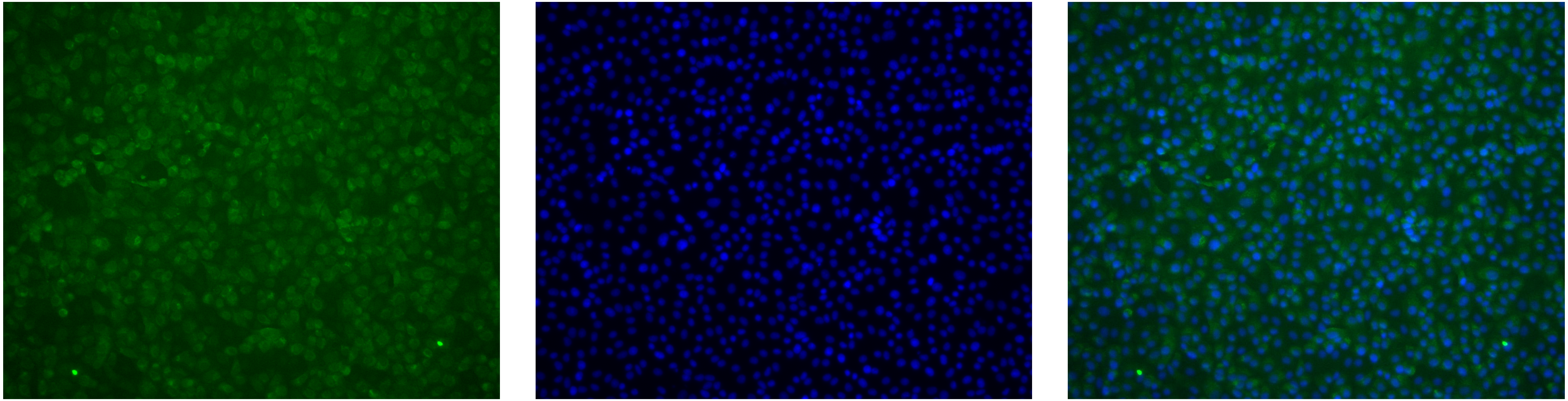

GFP DAPI Overlay

Figure 4B. 10 second

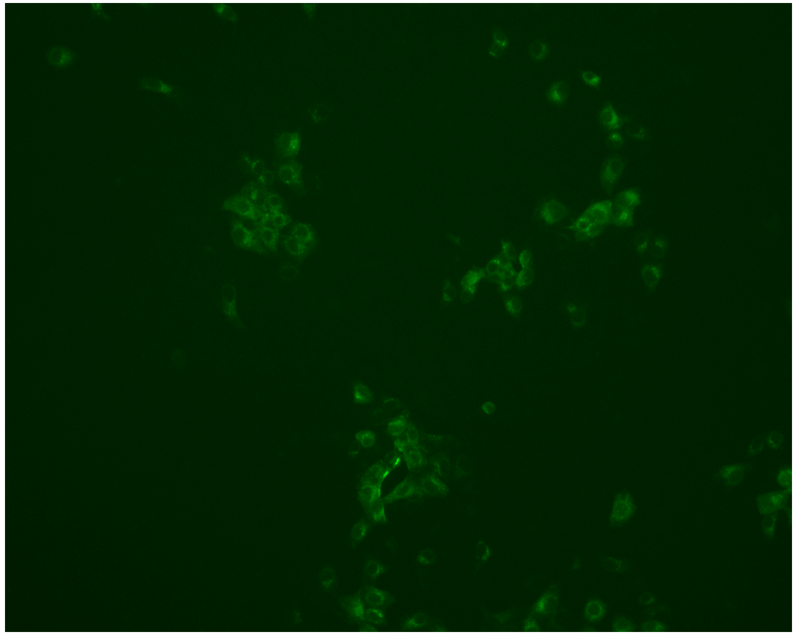

GFP

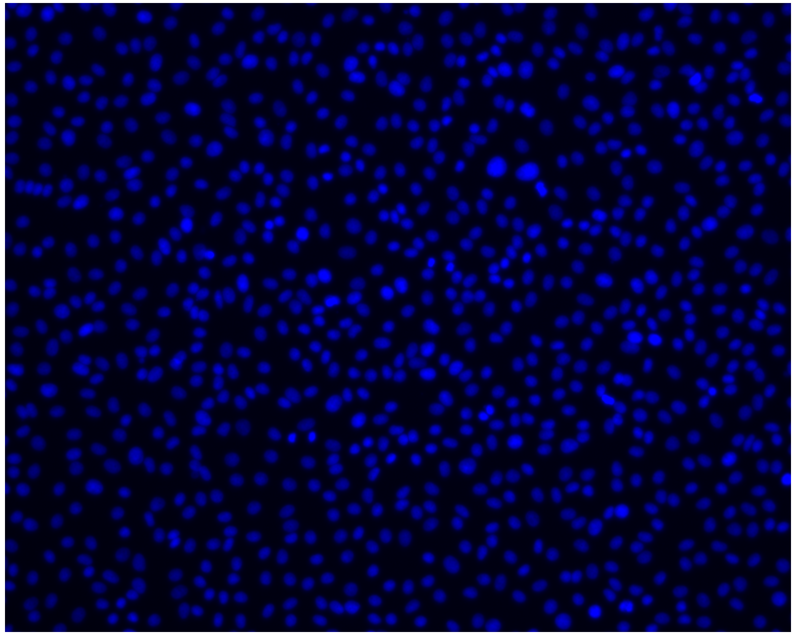

DAPI

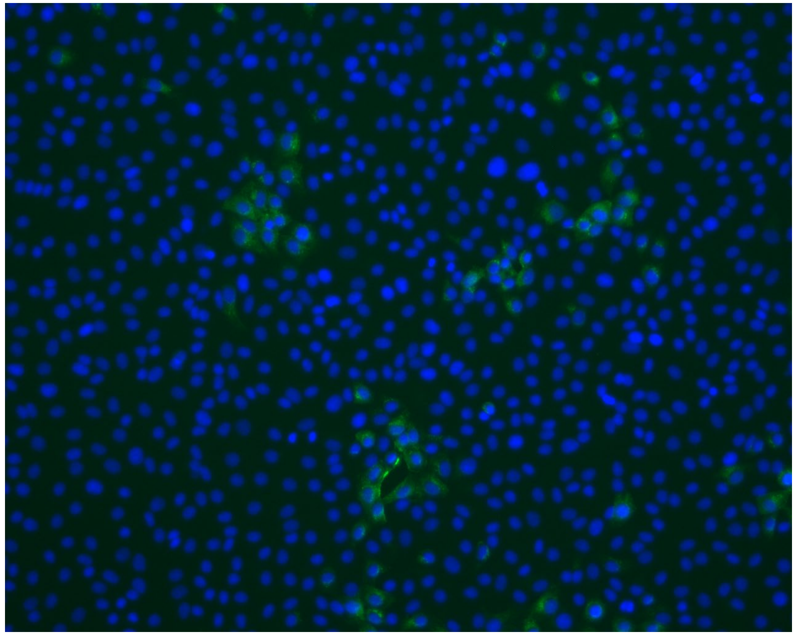

Overlay

Figure 4B. 30 second

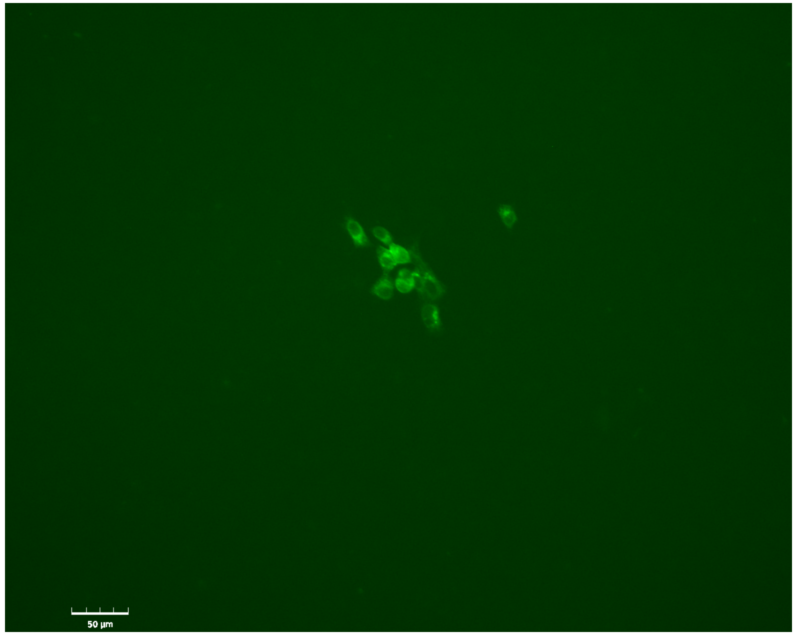

GFP

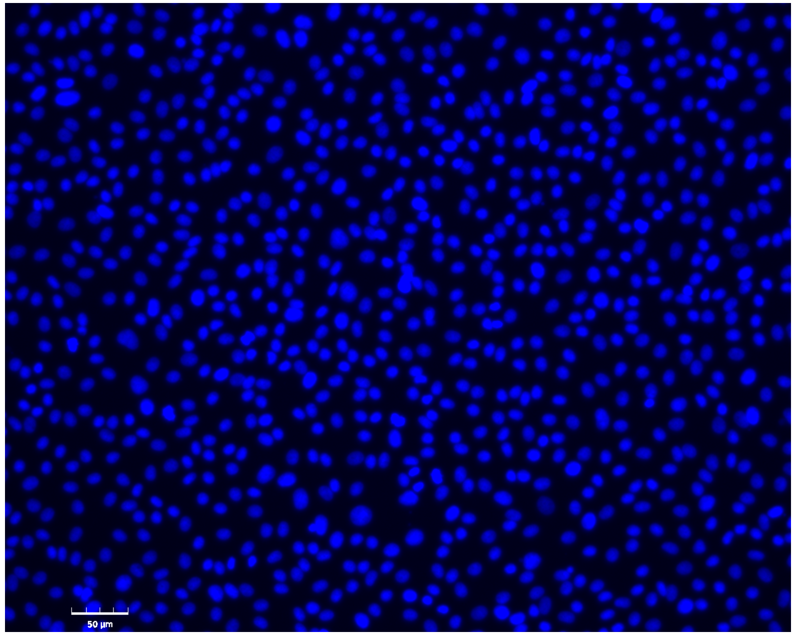

DAPI

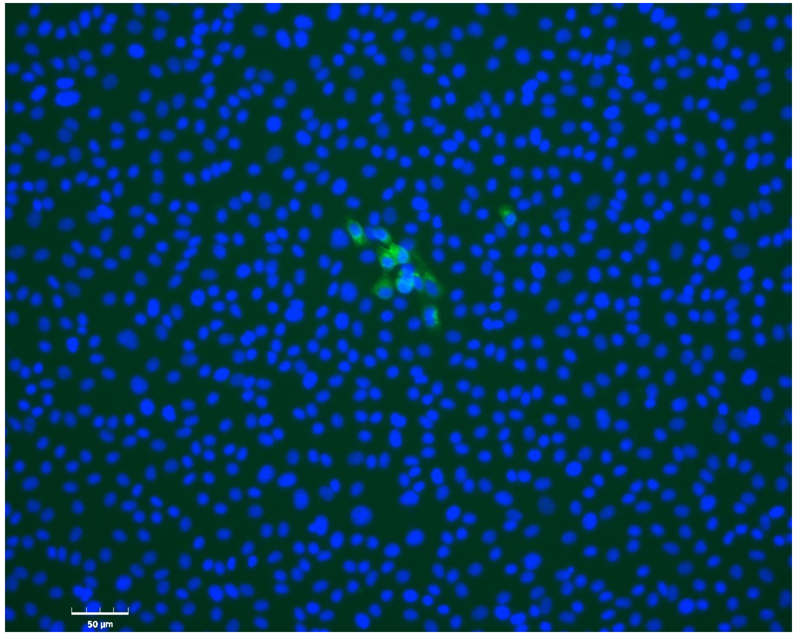

Overlay

Figure 4B. 60 second

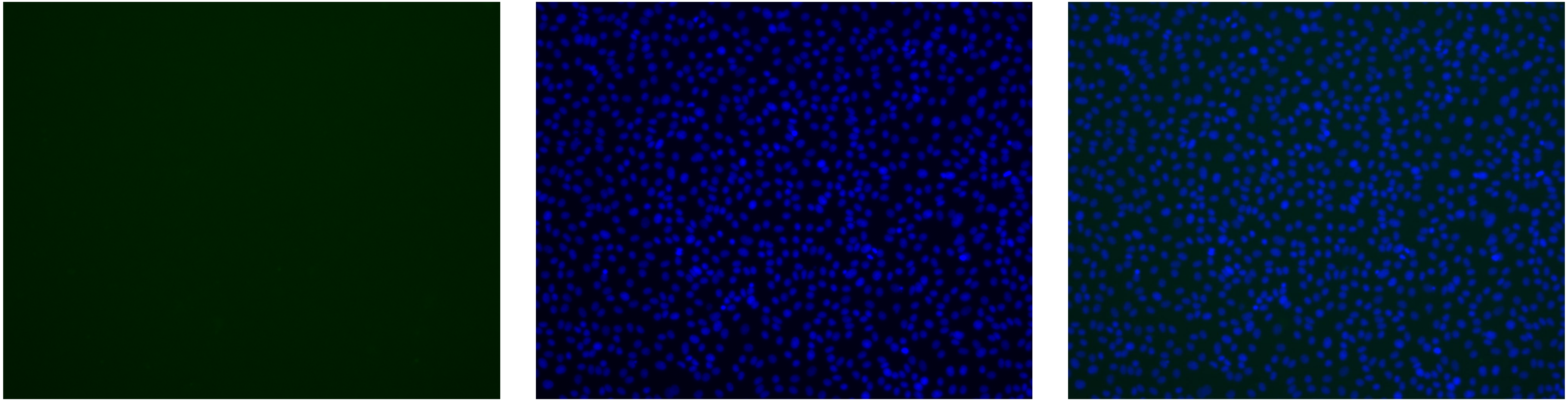

GFP DAPI Overlay

Figure 5A. B1.617.2 0 second

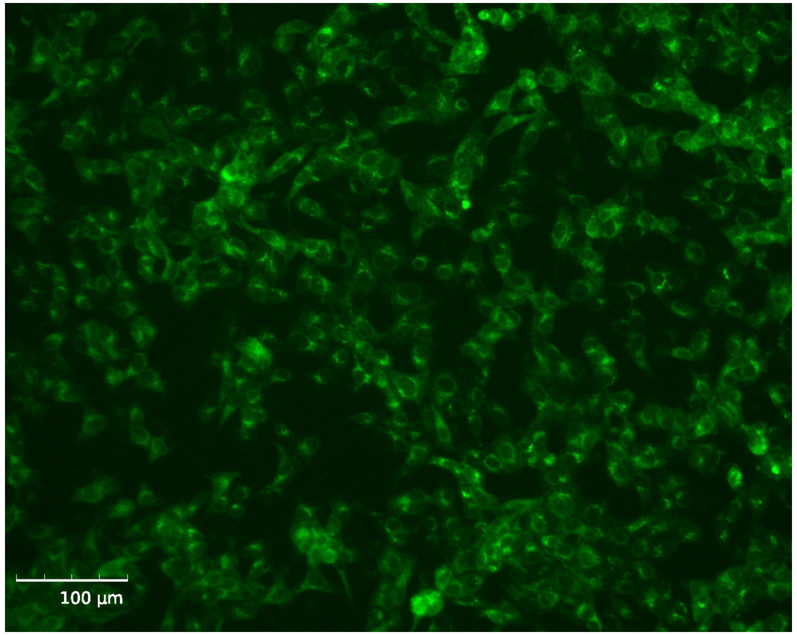

GFP

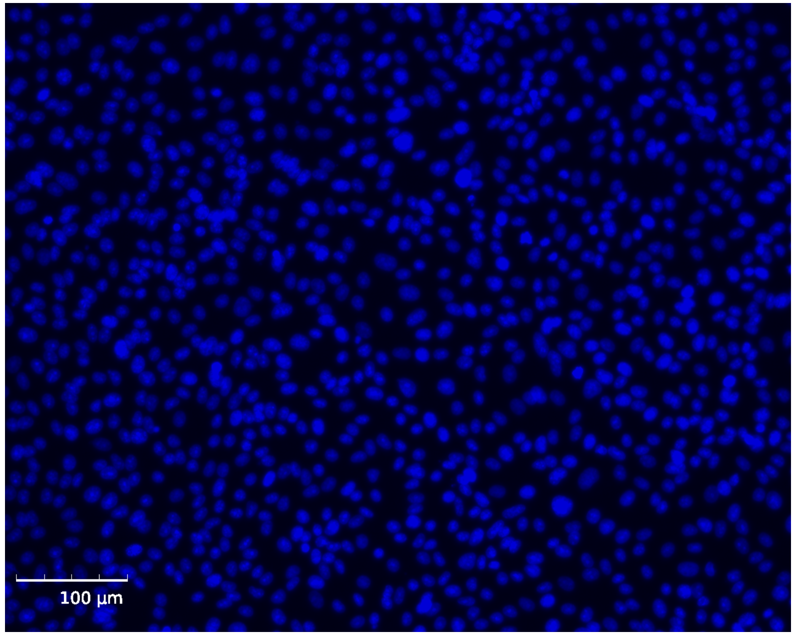

DAPI

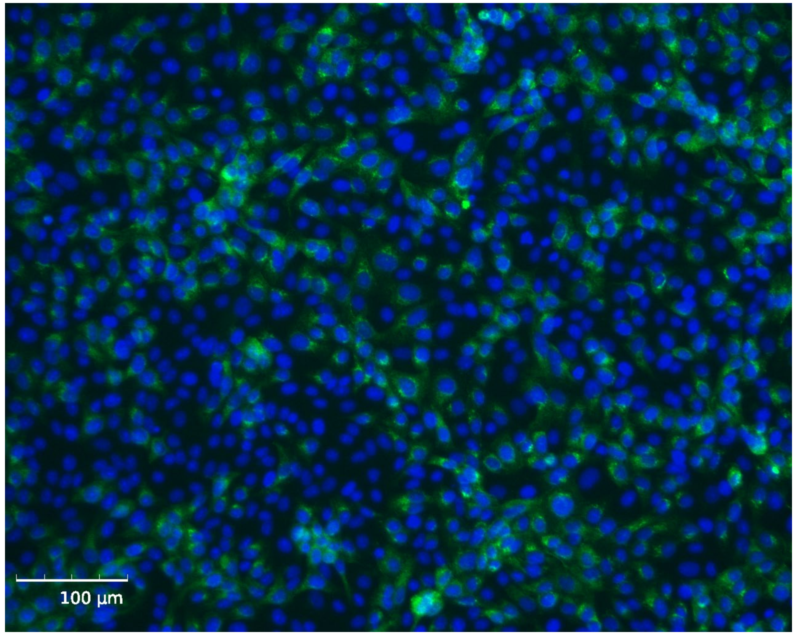

Overlay

Figure 5A. B1.617.2 10 second

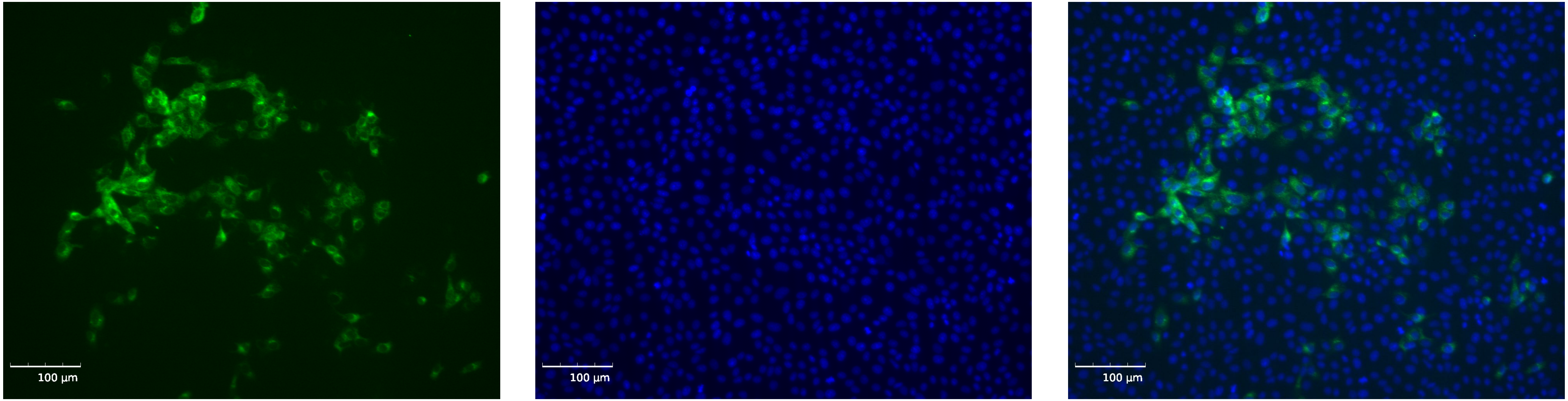

GFP DAPI Overlay

Figure 5A. B1.617.2 30 second

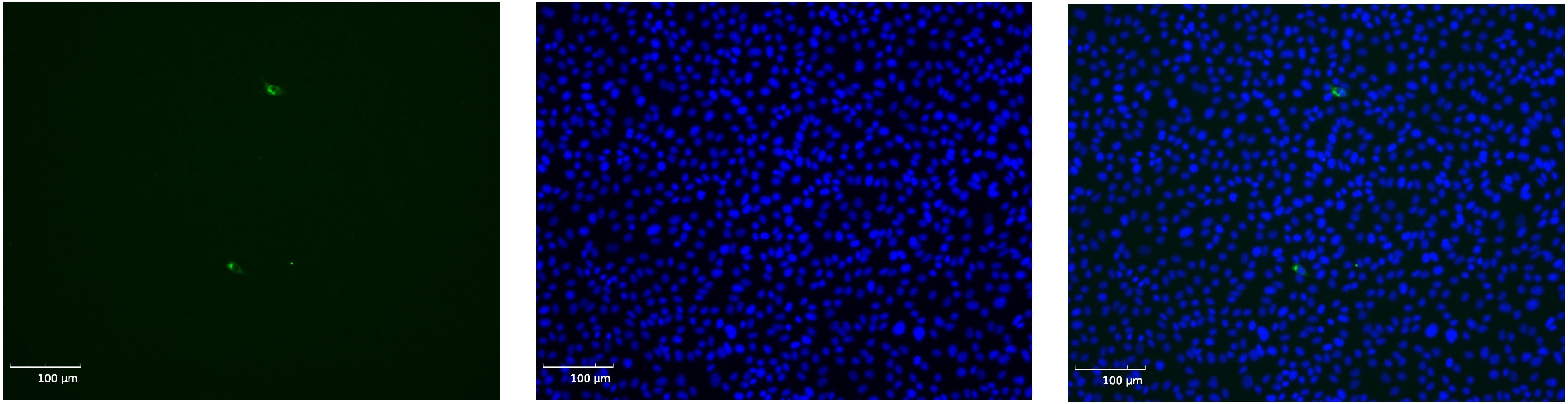

GFP DAPI Overlay

Figure 5A. B1.617.2 60 second

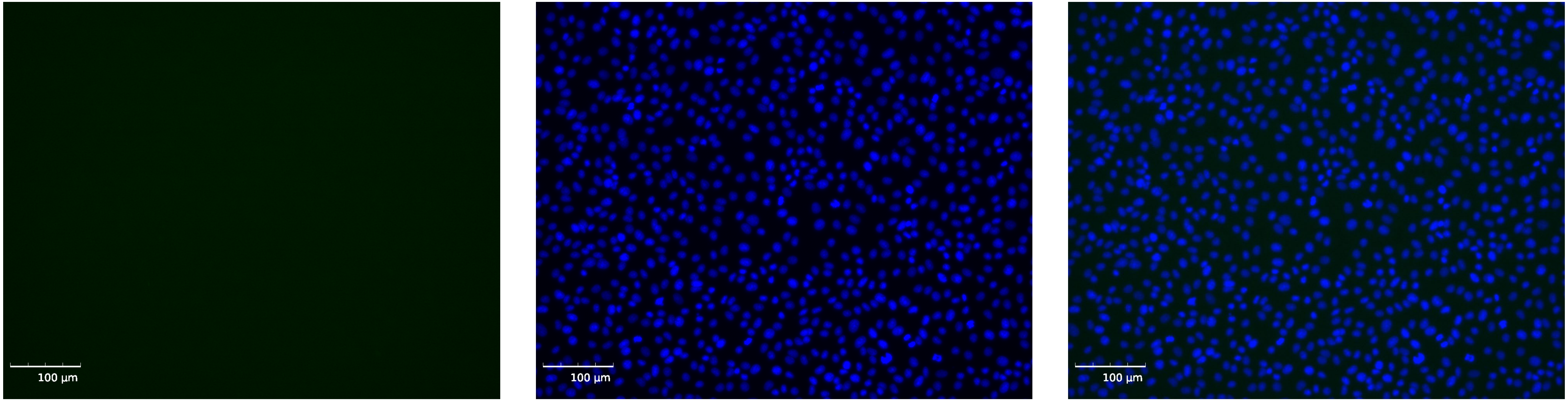

GFP DAPI Overlay

Figure 5A. BA.1 0 second

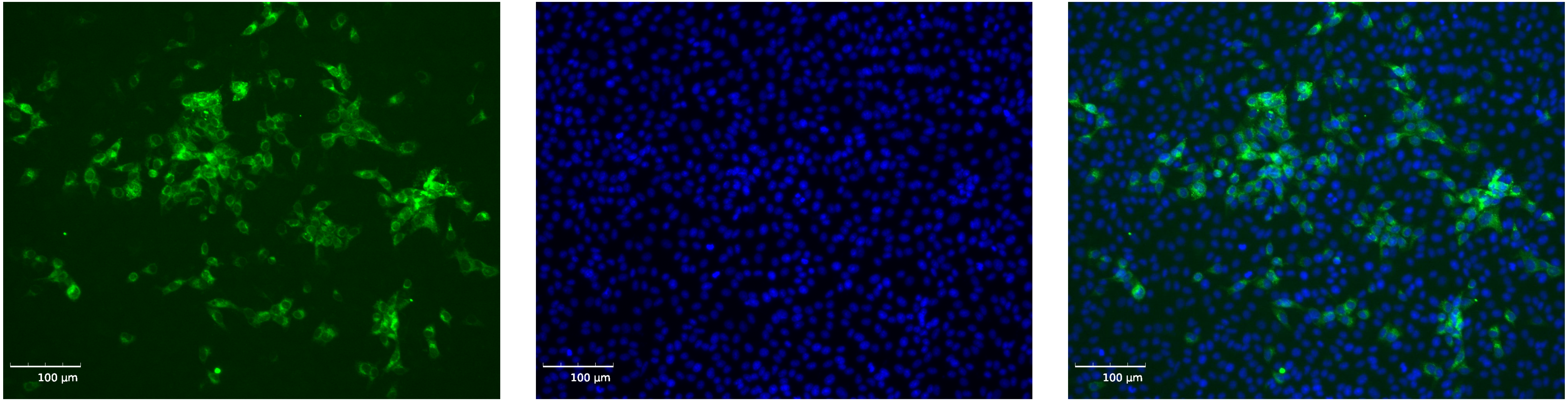

GFP DAPI Overlay

Figure 5A. BA.1 10 second

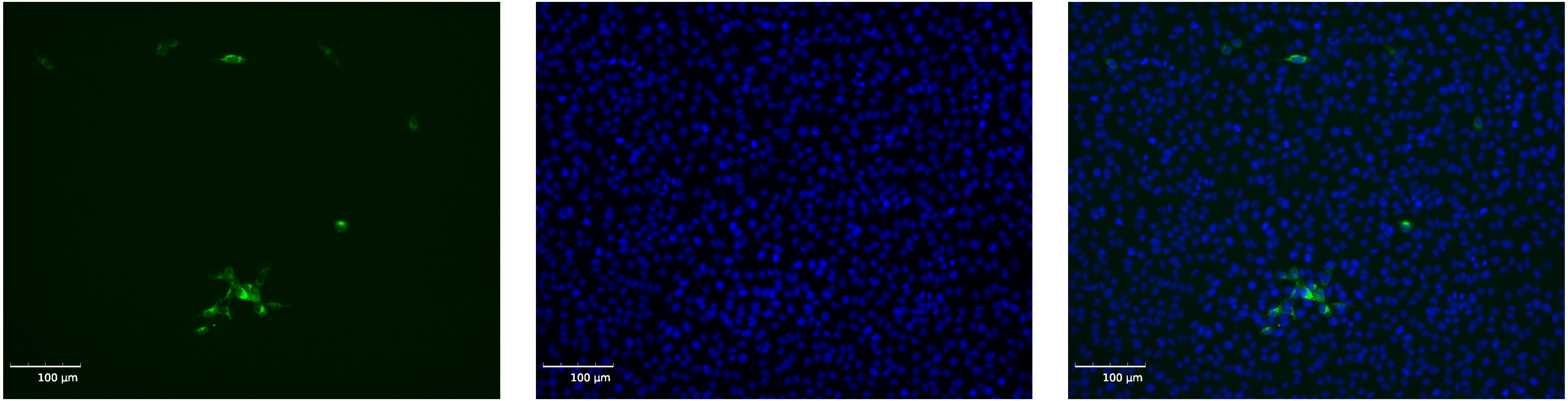

GFP DAPI Overlay

Figure 5A. BA.1 30 second

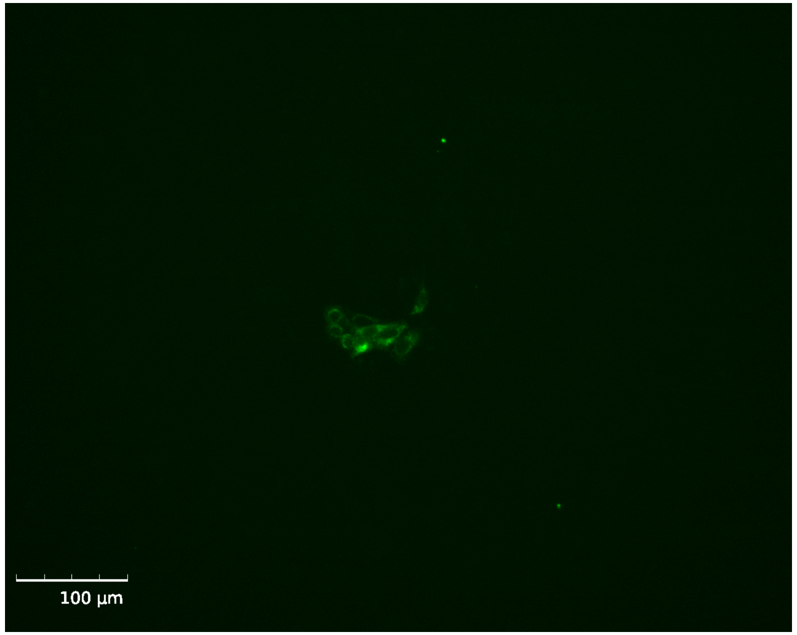

GFP

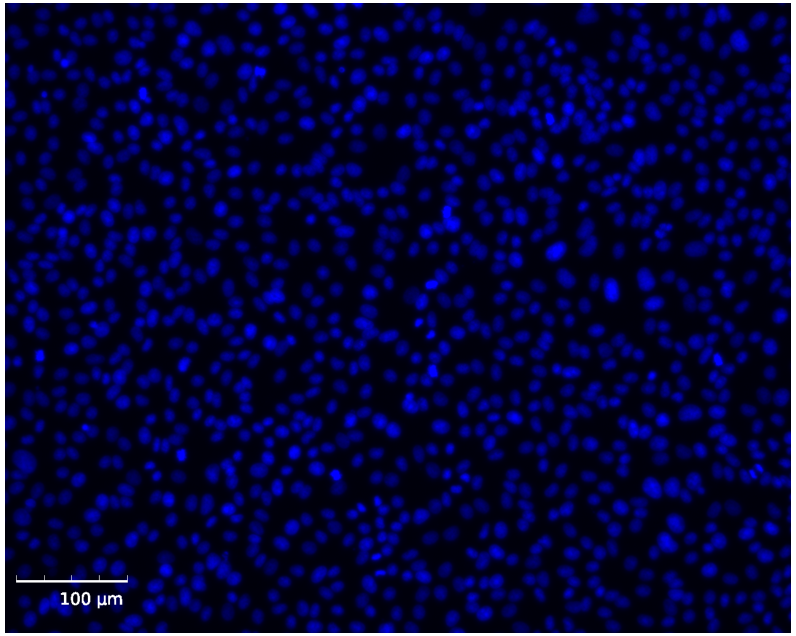

DAPI

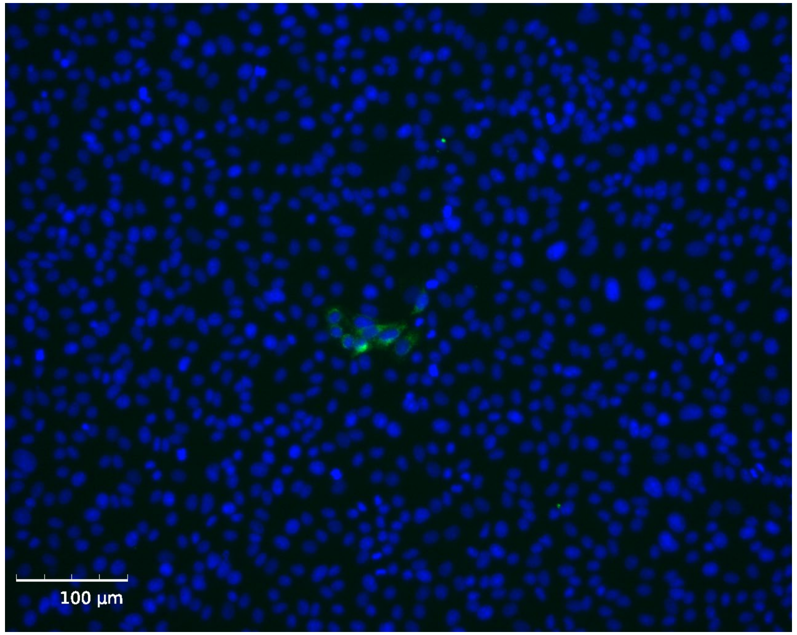

Overlay

Figure 5A. BA.1 60 second

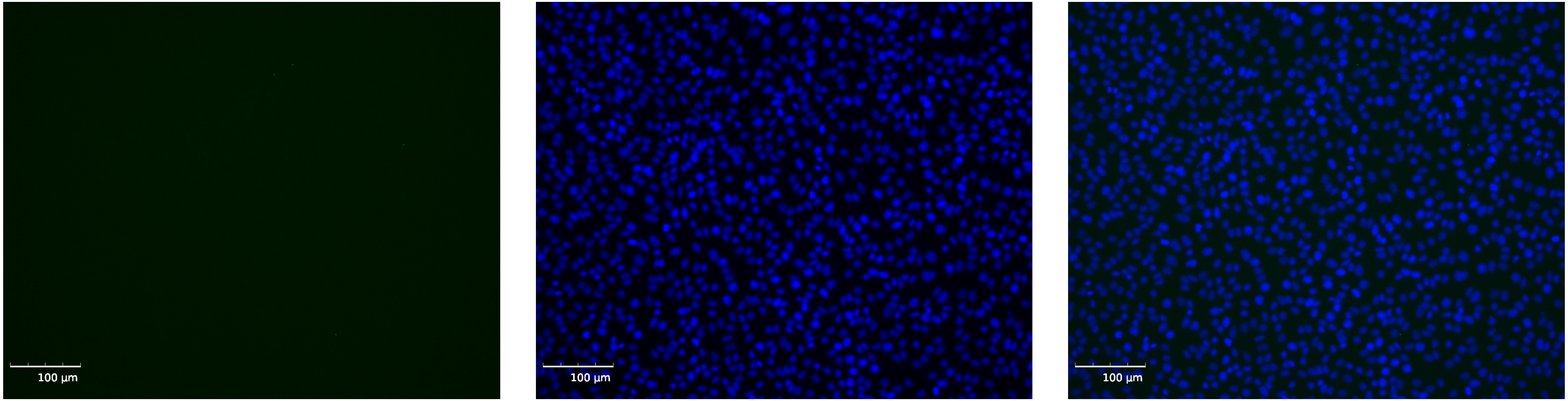

GFP DAPI Overlay

Figure 5B. S protein

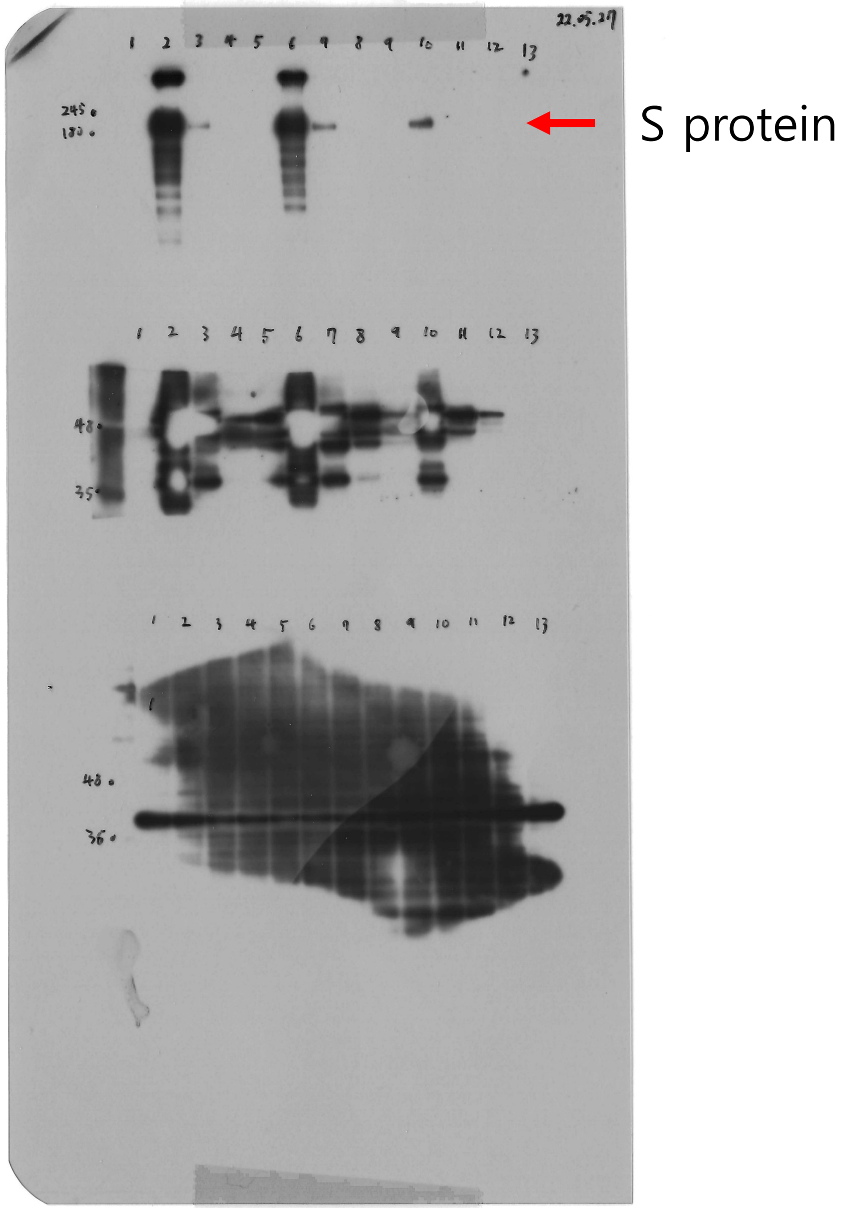

Figure 5B. N protein

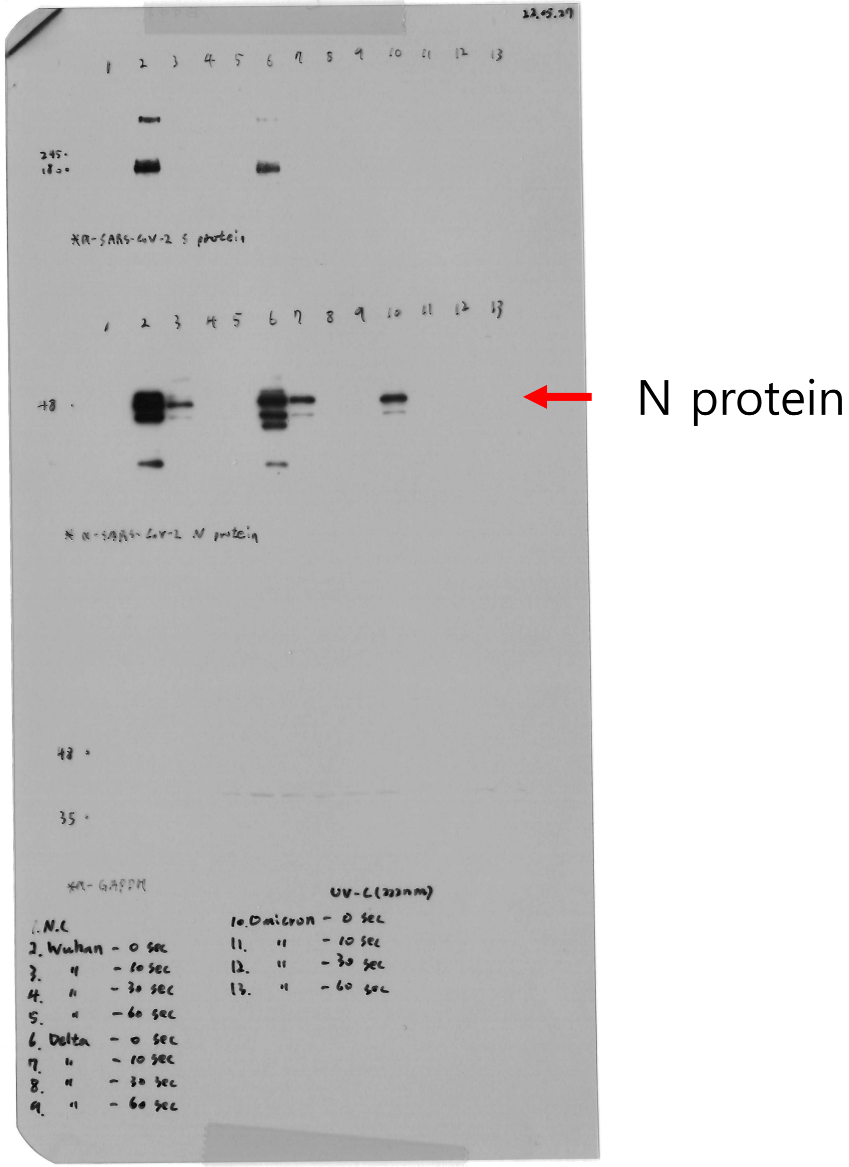

Figure 5B. GAPDH

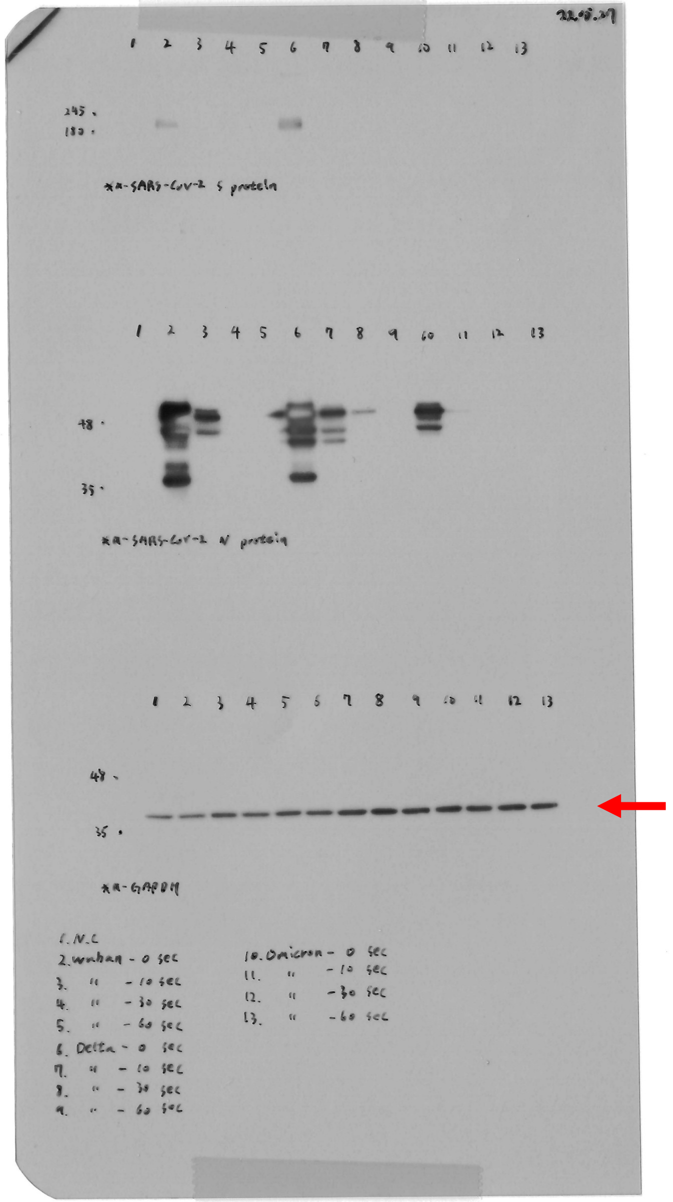

GAPDH
