# Supplementary material for: Ultraviolet-C light at 222 nm has a high disinfecting spectrum in environments contaminated by infectious pathogens, including SARS-CoV-2
Source: PLoS One. 2023 Nov 28;18(11):e0294427. doi: 10.1371/journal.pone.0294427 (PMC10684113; doi:10.1371/journal.pone.0294427)
Supplement: Attachment — Submitted filename: Response to Reviewers_SK.docx [file pone.0294427.s003.docx]

**Response to Reviewers**

Reviewer #1: In this manuscript, authors investigated the effect of UVC at 222nm for disinfection of pathogens, particularly SARS-Cov-2, comparing with 275nm UVC. Authors clearly showed that the 222nm UVC exerts the the germicidal and virucidal activity against SARS-cov-2 and couple of bacteria in various contaminating conditions (liquid, fabric) and can effectively inactivates SARS-cov-2 variants such as Delta and Omicron. Considering the significance of pandemic situation of infectious viruses such as Flu and Corona and versatility/safety of 222nm UVC, these data support the powerful preventive strategy against various pathogen. Nevertheless, the manuscript would be further strengthened if the authors can address the following minor concern.
1. In introduction section, author need to clearly clarify the aim of this study why they performed the experiment in liquid and fabric condition, not aerosol and compared the effect in various exposure time, etc. And it also needs to be discussed.

**Response) In the introduction section, I explained why we performed the experiments in liquid and fabric conditions in lines 60 – 62. Unfortunately, we did not have the equipment to measure aerosol spray distance, degree, and range in the BL3 facility, so we could not proceed with the disinfectant efficacy test of UV-C.**

Reviewer #2: This manuscript describes the use of Ultraviolet-C light at 222 nm has a high disinfecting spectrum in environments contaminated by infectious pathogens, including SARS-CoV-2 by Song. They examined the effect of 222 nm UV-C irradiation on contaminating bacteria and viruses. A colony forming unit assay was used to determine the number of viable cells, and a virus titration was used as the assay of evaluation. They also found that the irradiation effectively inactivated Delta and Omicron, variants of SARS-CoV-2. These results provide valuable information on the bactericidal efficiency of 222nm UV-C in environments contaminated with bacteria and viruses. The experimental methods, discussion, and argument of the paper are very well done.

**Response) I appreciate your positive comments.**

Reviewer #3: Authors demonstrated the disinfection efficiency of 222-nm UV-C in bacterial and virus-contaminated environments. Overall, the manuscript was conducted properly and well-written. And the results may provide the valuable information on disinfection. However, the finding lacks the novelty, because the disinfection efficiency of UV-C is already known. The authors did not describe the reason why that they selected 222-nm UV-C in their study. Generally, the disinfection efficiency of 222-nm UV-C is lower than that of 275-nm UV-C. There is no information supporting the safety and advantage of 222-nm UV-C. In virucidal study, the authors only tested enveloped RNA viruses. The authors may need to test other types of viruses such as non-enveloped or DNA viruses.

**Response) In the introduction section, I described the reason why we selected 222-nm UV-C in lines 54 – 57. The safety and advantage of 222-nm UV-C are already well known (reference 10, and 34), so this study did not test it. We have not secured non-enveloped RNA viruses or DNA viruses yet, indeed. However, 222-nm UV-C would be effective for non-enveloped RNA viruses as it penetrates the enveloped RNA viruses and shows disinfection activity. In the subsequent study, as you mentioned, we would like to conduct the sterilization effect of 222-nm UV-C on the non-enveloped RNA virus or DNA viruses.**
